# Indents

**Published:** 1904-02-15

**Authors:** 


### INDENTS.

**☞ Brief Articles of Technical or Scientific Value will receive notice and comment. Contributions invited.**

**Case Hardening.** Heat the piece to be hardened, with a blowpipe until it has become cherry red in color and keep dusting on ferro cyanid of potash for a few minutes. Heat up again and plunge into cold water. If the metal comes out grayish white in color no file can even scratch it, but if it does not the cyanide must be kept on longer.—J. Mills, in *Review.*

**"Cleaning Teeth."** This is a beastly term and it should be relegated to oblivion. Suppose a patient should apply to the otologist to have his ears "cleaned," or to the rhinologist to have his nose "cleaned," what might not happen, especially if the doctor happened to be the bigger man? But the dental patient is not to blame, and this fact should make the would-be offended dentists considerate indeed. We have advised, recommended, charged for "cleaning teeth" for a century; we have educated the patient beyond the possibility of "Lest we forget," and like good chickens, this and kindred terms naturally come daily home to roost. In the interest of reform and the habitual use of befitting terms, let us see to it that the patient, by means of brush, powder, washes, silk and rubber bands, disposes of all the dental refuse before coming to the office, leaving for us the purely professional task of "removing salivary and other deposits," "treating mechanically and medicinally the gums," "putting teeth and conjoined parts in a healthful condition." By all means let us quickly get out of our daily vocabulary terms that best become the dental scavenger.—*Dental Office and Laboratory.*

**Septicemia from Alveolar Abscess.** Dr. Morris makes the statement that many patients die from septic pneumonia due to the extraction of an abscessed tooth. He advised doing no work when infection is in active prolification. He complicates the matter from a dental standpoint by stating that that condition can only be determined by slides.

The application of heat or ice in such conditions of infection should be in accordance with one's judgment. Tonics should be administered so as to increase cell resistance.

In evacuating an abscess the tendency is to use pressure. Don't do it, for then an abscess is likely to run its best course. It is liable to spread the infection by pressure. The use of pyrozone in sterile wounds is liable to produce new infection. Don't wash out an abscess. Don't disturb it, nature's albuminous substance is a very bland thing to have in there.—*Review.*

**Pink Rubber Fronts.** In David Christy Murray's novel of "Valentine Strange," he makes Hiram Search say: "How pretty a piece of work looks when one's done it one's self!" Well, Hiram, my lad, that's because, consciously or unconsciously, one puts a bit of one's self into every piece of work one does. If we could keep this thought constantly before us in all our operations, and put only the best of ourselves in each operation, the best would, with each succeeding attainment, become better. With such a thought in mind would we not hesitate to put pink rubber in a conspicuous place in the mouth, where in a few months we shall meet it in the form of a *faded* smile? May we be forgiven for the smiles we have *ruined.* What is there that has done more to enlighten the world's heartaches and burdens than smiles, smiles that are the sunshine of the soul? Pass the smiles along in a good, wholesome way. Let them come as a reflex to our own good fellowship. How we cherish the smile of a friend! But deliver us from ever having to meet one with a pink rubber front. It gives us the shivers and doubly so if we know we are the guilty one who is responsible for the handicap. Guilty every one of us! May we be less and less so as the days go by.—Dr. Hetrick, in *Western Journal.*

**Cheap Amalgam Fillings.** Of all materials used by the dentist, amalgam is considered the cheapest, and so it is inserted in the cheapest way possible. But the first and great trouble, according to my opinion, is the idea of so-called "cheap work." I have practiced dentistry thirty-seven years, and I am willing to go on record as saying that there is not a dentist in the United States who can and does put in a first-class amalgam filling for one dollar. Yet, that is the average price all over the country. I have always gotten two dollars for every plain amalgam filling I ever put in, and have always been so poor that I have been compelled to "lean on the fence to gobble"—and why? The simple fact is, that I put from three to five times more work on an amalgam filling that the average dentist does, and I think, and really know, that that is the secret of the life of amalgam fillings. I have numbers of amalgam fillings that have been in thirty years. I have hundreds that have been in twenty years. Then why is it that the average life of amalgam fillings is only four years? I believe I can tell you. The average dentist puts in amalgam fillings as a cheap material, and as he gets only one dollar per filling for his work, he buys the very cheapest amalgam on the market. Consequently, he gets results such as I saw a short time since. A man came into my office who had some teeth filled a few days before. The fillings were worn out and he had violent toothache. He said he had had them filled last week, but in going home his horse had jolted the fillings out and the teeth had been aching ever since. We laughed at the idea, when he remarked, "I'll swear to God it's so." Well, according to my notion on the subject, it is the dentist's fault the amalgam fillings do not last longer. As I have said before, dentists are born, and not made. But even those who are born dentists can't put in first-class fillings with fifth-class amalgam for one dollar a filling.—Geo. S. Staples, in *Texas Journal.*

**Practice Building.** A new wrinkle in the art of securing a practice has just come to the notice of the writer, and it is here recorded for the benefit of those who may find it a slow process getting patients. It is mentioned as a good example of how not to do it. A lady book agent was in the habit of calling at houses and offering her books for sale. After canvassing the household in a literary way her talents took a literal turn, and she ask if any of the family had dental work to be done. She said that her son had just opened an office, and that she would be glad to secure patients for him among those upon whom she called to sell books. In other words, while she was anxious to fill their brains with knowledge, she was fully as anxious that her son should fill their teeth.

Now, it is the farthest thing imaginable for me to censure this good mother for what she did. It was probably done with the best of motives and from the boundless love she bore that boy. She could not be expected to know much about the ethics of professional life, and as for the abstract propriety of such a procedure—well, it was *her* boy, and she wanted to help him. But with the boy himself it is different. No young man can go through college to-day and enter practice without learning enough of the ethics of the profession to make him aware of the essential quackery of such a method.

What he should have done was to put his arm around his mother and tell her not to do that. If she asked the reason, he could say to her that such a procedure would compromise his reputation, not only with the profession, but with the very class of people whom he would ultimately wish to win as patients. It is safe to say that no substantial practice was ever built upon such a basis as that, and the sooner young practitioners learn this the better it will be for them. I trust that this young man has headed his mother off before now, but if not, he may be assured that he will soon be the laughing-stock of the community.—*Review.*

**Artist and Artisan.** Practitioners of dentistry should be artists, but there are, unfortunately, a a few who are merely artisans. These latter do their work in a mechanical way, being merely skilled in mechanical art. They have been told or shown that certain work should be done thus and so; and thus and so it is done without any apprehension of the why and the wherefore. They labor according to rule, and the facts are unconsidered. Eyes have they, but they see not; ears have they, but they hear not; brains have they, but they think not.

Good taste will suggest in dentistry the arrangement of prosthetic pieces after the slight irregularities generally found in nature and according to the best cosmetic effect, in preference to the regularity of unthinking artificiality. Science, guided by its knowledge of truths and general principles ascertained by observation, experimentation and deduction, will suggest the best arrangement to withstand masticatory stress and occlused strains.

The artisan, however, devoid of either, may be expected to set up teeth with the same stiff regularity he would evidence in his work were he at his proper avocation of building a picket fence. When he has taken an exact impression and made a plate on the exact model taken therefrom, he wonders why the product of his labor is a misfit.

The artist, on the other hand, takes cognizance of, and scientifically makes allowance for, the soft and hard palatal areas, etc., and accordingly has a gratified patient.

The artisan anneals his gold (to save expense) with fusel *oil* or water gas, and wonders why the filling gold he procures is so poor in quality.

The scientist, knowing that mechanical "cleanliness is next to godliness," in manipulating filling gold, uses ethyl alcohol or electric annealers and zealously guards against foreign matters interfering with the welding of his gold.

Unfamiliarity with the laws of natural and physical science has caused many experimental failures in all lines of life, and largely reduced the value of many works of art.—*Stomatologist.*

**In Defense of a Tooth Saver.** In the *Items of Interest* for December, Dr. W. Farley, Paris, France, describes a method of banding "back" teeth in order to save them *permanently*, and in the course of his article says: "It is estimated that the life of an amalgam filling will average four years. Also, that four-fifths of all fillings are amalgam. Amalgam, then, can not be called a permanent filling. It is not serious enough for the pretenses of a profession. It is more a barber's material when employed in such cases. This will not do. Let us spend less time in discussing ethics and more time in discussing the preservation of ordinary teeth, which, heretofore, have evaded our boasted skill. If I were addressing a room full of students, I would tell them that a tooth which is decayed below the gum margin cannot be preserved with a plastic filling, seldom with gold, *but* as a rule, always with a band."

As to banding teeth to repair the ravages of decay, we feel there is little to be said; the operation is, in the majority of cases, impossible of successful performance, and in our opinion, will almost invariably prove a failure, particularly if in fitting the band the tooth is ground "sparingly" as the author directs. A band of any kind placed on a tooth for any purpose, must, in order to be clean and unirritating, fit the tooth *accurately* as its cervical border.

In any tooth inclined to be bell-shaped, this can be accomplished only by grinding away its convex surfaces until it assumes a cylindrical form. To do this destroys the contour of the tooth, and not to do it insures an ill-fitting band. This would be most particularly the case when the band was intended to pass deeply under the gum, as in covering a deep cavity.

Even if a band could be fitted accurately in such a case the disintegration of the cement with which it is set will, in the course of time, surely tend to its failure.

It is certain that where a tooth has reached such a stage of decay that it cannot be successfully filled, one of the many forms of crowns, wisely selected and skillfully placed in proper position, will afford the best means of saving it.

As to the author's remarks about amalgam, one can but wonder after reading them, what sort of amalgam work he has seen, and what kind of amalgam he has used in his own practice, and how he handled it, in order to reach such conclusions.

If there is any one place where amalgam, even when used with but moderate skill, has been an unqualified success it is in deep cavities, extending far under the gum in almost hopeless teeth. It is in such cases that amalgam has no rival as a tooth saver, and where it should be used to the exclusion of every other filling material, with the possible exception of gutta-percha, which, of course, cannot be used where it must bear any great stress of mastication.

Amalgam has been decryed for years, and though misused, abused and neglected, has yet saved thousands of teeth *permanently*. It is not the material but its abuse which has led the author of the article quoted to reach such erroneous conclusions. When dentists have learned to charge such a fee for their services as will permit them to do their work properly and thoroughly, regardless of the material employed, or its cost, there will be fewer amalgam fillings placed in but partially prepared cavities, and left with rough and protruding margins and unpolished surfaces. Then, too, will this material receive just consideration for its many virtues and inestimable value as a tooth saver.—Editorial in *Pacific Gazette*.

